# Targeting Akt/NF-κB/p53 Pathway and Apoptosis Inducing Potential of 1,2-Benzenedicarboxylic Acid, Bis (2-Methyl Propyl) Ester Isolated from *Onosma bracteata* Wall. against Human Osteosarcoma (MG-63) Cells

**DOI:** 10.3390/molecules27113478

**Published:** 2022-05-28

**Authors:** Ajay Kumar, Sandeep Kaur, Sukhvinder Dhiman, Prithvi Pal Singh, Gaurav Bhatia, Sharad Thakur, Hardeep Singh Tuli, Upendra Sharma, Subodh Kumar, Abdulmajeed G. Almutary, Abdullah M. Alnuqaydan, Arif Hussain, Shafiul Haque, Kuldeep Dhama, Satwinderjeet Kaur

**Affiliations:** 1 Department of Botanical and Environmental Sciences, Guru Nanak Dev University, Amritsar 143005, India; ajaybot.rsh@gndu.ac.in (A.K.); soniasandeep4@gmail.com (S.K.); 2 Department of Chemistry, Guru Nanak Dev University, Amritsar 143005, India; sukhvinderdhimank@gmail.com (S.D.); subodh_gndu@yahoo.co.in (S.K.); 3 Chemical Technology Division, CSIR-IHBT, Palampur 176061, India; thakurprithvi028@gmail.com (P.P.S.); upendra@ihbt.res.in (U.S.); 4 Academy of Scientific and Innovative Research (AcSIR), Ghaziabad 201002, India; 5 Department of Biochemistry, Pt. Jawaharlal Nehru Government Medical College and Hospital Chamba, Chamba 176310, India; gauravbhatia22@gmail.com; 6 Biotechnology Division, COVID-19 Project, CSIR-IHBT, Palampur 176061, India; thakursharad23@gmail.com; 7 Department of Biotechnology, Maharishi Markandeshwar (Deemed to be University), Mullana, Ambala 133207, India; hardeep.biotech@gmail.com; 8 Department of Medical Biotechnology, College of Applied Medical Sciences, Qassim University, Buraydah 52266, Saudi Arabia; ami.alnuqaydan@qu.edu.sa; 9 School of Life Sciences, Manipal Academy of Higher Education, Dubai Campus, Dubai 345050, United Arab Emirates; dr.arifhussain@yahoo.co.in; 10 Research and Scientific Studies Unit, College of Nursing and Allied Health Sciences, Jazan University, Jazan 45142, Saudi Arabia; shafiul.haque@hotmail.com; 11 Bursa Uludağ University Faculty of Medicine, Görükle Campus, 16059 Nilüfer, Turkey; 12 Division of Pathology, ICAR-Indian Veterinary Research Institute, Bareilly 243122, India; kdhama@rediffmail.com

**Keywords:** antiproliferative activity, apoptosis induction, G_0_/G_1_ phase, *Onosma bracteata*, osteosarcoma, ROS

## Abstract

*Onosma bracteata* Wall. is an important medicinal and immunity-enhancing herbs. This plant is commonly used in the preparation of traditional Ayurvedic drugs to treat numerous diseases. Inspired by the medicinal properties of this plant, the present study aimed to investigate the antiproliferative potential and the primary molecular mechanisms of the apoptotic induction against human osteosarcoma (MG-63) cells. Among all the fractions isolated from *O. bracteata*, ethyl acetate fraction (Obea) showed good antioxidant activity in superoxide radical scavenging assay and lipid peroxidation assay with an EC_50_ value of 95.12 and 80.67 µg/mL, respectively. Silica gel column chromatography of ethyl acetate (Obea) fraction of *O. bracteata* yielded a pure compound, which was characterized by NMR, FTIR, and HR-MS analysis and was identified as 1,2-benzene dicarboxylic acid, bis (2-methyl propyl) ester (BDCe fraction). BDCe fraction was evaluated for the antiproliferative potential against human osteosarcoma MG-63, human neuroblastoma IMR-32, and human lung carcinoma A549 cell lines by MTT assay and exhibited GI_50_ values of 37.53 μM, 56.05 μM, and 47.12 μM, respectively. In MG-63 cells, the BDCe fraction increased the level of ROS and simultaneously decreased the mitochondria membrane potential (MMP) potential by arresting cells at the G_0_/G_1_ phase, suggesting the initiation of apoptosis. Western blotting analysis revealed the upregulation of p53, caspase3, and caspase9 while the expressions of p-NF-κB, p-Akt and Bcl-xl were decreased. RT-qPCR studies also showed upregulation in the expression of p53 and caspase3 and downregulation in the expression of CDK2, Bcl-2 and Cyclin E genes. Molecular docking analysis displayed the interaction between BDCe fraction with p53 (−151.13 kcal/mol) and CDK1 (−133.96 kcal/mol). The results of the present work suggest that the BDCe fraction has chemopreventive properties against osteosarcoma (MG-63) cells through the induction of cell cycle arrest and apoptosis via Akt/NF-κB/p53 pathways. This study contributes to the understanding of the utilization of BDCe fraction in osteosarcoma treatment.

## 1. Introduction

Cancer is a complicated disease in which cells multiply and grow uncontrollably due to altered signaling pathways in different tumor types. Osteosarcoma (OS) is a primary malignant bone sarcoma that occurs in children and adolescents, with ~10% of OS occurring in individuals older than 60 years [1]. Corre et al. [2] reported the occurrence of osteosarcoma as 1 to 3 cases per million within 15–19 years of age annually. OS generally develops via cell proliferation perturbation, dysregulation of cell cycle, and mutations in DNA, and around 70% of osteosarcoma cases showed chromosomal aberrations [3]. Existing chemo-therapeutic medication does not show a significant effect in combating cancer [4,5]. Although there are a variety of modern treatment strategies available for treating cancer, these strategies still have limitations such as re-occurrence, metastasis, low success rate, and several side effects [6,7]. The cancer cells undergo incessant mutations that reduce the effectiveness of cancer-targeting approaches [8]. Cancer cells can be metastasized to distant parts of body organs, even after the tumor is completely removed from the primary site [9]. Therefore, there is an urgent need to develop effective treatment strategies to prevent cancer. The involvement of the Akt, NF-κB, p53, and numerous signaling pathways in cancer has been studied extensively [10]. Different types of cancer show the activation of the Akt, NF-κB pathway and the inactivation of the p53 pathway [11]. In around half of all human cancer, p53 gene mutations are most prevalent [12]. In cancer cells, the restoration in the activity of these genes could be an appealing therapeutic option. Therefore, considering the above-mentioned circumstances, the challenge is to selectively eliminate cancer-promoting cells via tumor-specific biomarkers that are involved in carcinogenesis. Medicines that target these pathways are unlikely to be effective in the treatment of a wide range of malignancies. For the past few decades, a tremendous body of research has recognized natural compounds isolated from plants as valuable sources of drugs, especially for cancer treatment [13]. Around 60% of anticancer drugs, explored clinically, have been isolated from natural products [14]. Phytoconstituents exhibited antioxidant and anticancer properties via molecular mechanisms involved in targeting receptors and enzymes present in signal transduction pathways associated with cell proliferation (Bcl-2), inflammation (NF-κB), apoptosis (p53, caspases), and multidrug resistance [15,16]. *Anchusa italica* (Boraginaceae) showed various types of biological activities that have been attributed to the presence of phytoconstituents in it [17].

*Onosma bracteata* Wall. (Boraginaceae) is an important medicinal herb that is largely found in high-altitude areas of India and Nepal [18]. It is used in the synthesis of various drugs in Unani and Ayurvedic medicinal systems due to its beneficial health effects [19]. In the indigenous system of medicine, the flowers, leaves, and seeds are used widely as cooling, acrid, and antipyretic substances and are also recommended in chest and lung diseases, throat troubles, stomatitis and, asthma [20]. *O. bracteata* has been shown to possess various types of pharmacological properties [20,21,22]. Albaqami and coworkers [22] reported the caspase-3 activation, lipid peroxides (MDA) inhibition, and anti-proliferative activity of *O. bracteata* in breast cancer (BT549), prostate cancer (PC3), and lung cancer (A549) cells. Ethyl acetate fraction (*Obea*) from *O. bracteata* was demonstrated to effectively inhibit the proliferation of MG-63 cells [23]. The present study plans to isolate the phytoconstituent(s) responsible for the anticancer potential and aims at the isolation of effective active compound(s) from *Obea* fraction with excellent anticancer potential. Silica gel column chromatography of *Obea* fraction yielded 1,2-benzene dicarboxylic acid, bis (2-methyl propyl) ester, which possesses potent anti-proliferative activity and was further studied for its role in the induction of apoptosis in MG-63 cells. This is the first study to report the anticancer potential of BDCe fraction isolated from *O. bracteata* against osteosarcoma.

## 2. Results

### 2.1. Antioxidant Activity

#### 2.1.1. Superoxide Anion Radical Scavenging Assay

**Figure 1:**
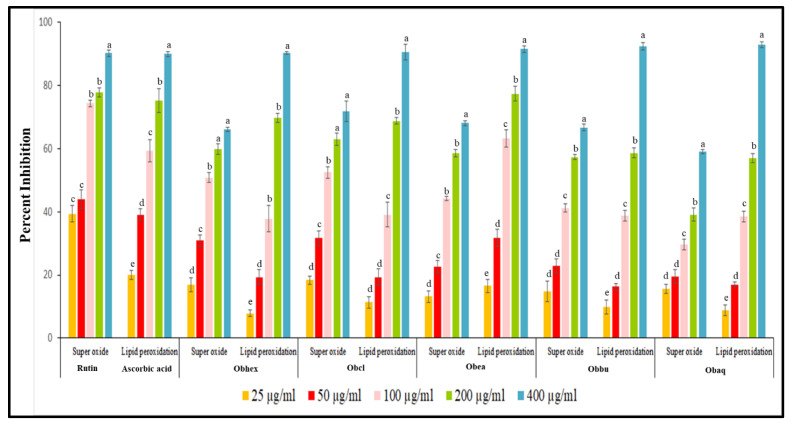
Antioxidant potential of various fractions obtained from *O. bracteata* using superoxide anion radical scavenging assay and lipid peroxidation assay. Results showed Mean ± SE of performed three experiments independent in triplicates. Data labels with different letters represent significant difference among them at (*p* ≤ 0.05).

Among all the fractions of *O. bracteata*, the Obea showed effective radical-scavenging potential (Figure 1) followed by chloroform fraction (Obcl). As shown in Supplementary Table S1, the EC_50_ values of Obea, Obcl, and Rutin on scavenging superoxide radical were 95.12 µg/mL, 114.30 µg/mL, and 46.18 µg/mL, respectively. At 400 µg/mL, the percentage inhibition of the Obea was 85.36 ± 2.60, and that of Rutin was 90.16 ± 1.07. 

#### 2.1.2. Lipid Peroxidation Assay

The *Obea* exhibited maximum potential to inhibit lipid peroxidation (Figure 1) with lower EC_50_ value of 80.67 µg/mL as compared to EC_50_ value of 76.77 µg/mL of the reference compound, Rutin (Supplementary Table S2). The *Obea* has a potent lipid inhibition percentage of 16.50 ± 2.10 at 25 µg/mL concentration, whereas Rutin has 20.06 ± 1.36 percent inhibition. The Obcl showed effective lipid peroxidation potential with EC_50_ value of 114.31 µg/mL followed by Obhex with EC_50_ value of 117.52 µg/mL. Therefore, keeping in mind their antioxidant potentials, *Obea* was further subjected to column chromatography.

### 2.2. Identification and Isolation of Bioactive Compound

#### 2.2.1. Isolation of Pure Compound from Silica Gel Chromatography

**Figure 2:**
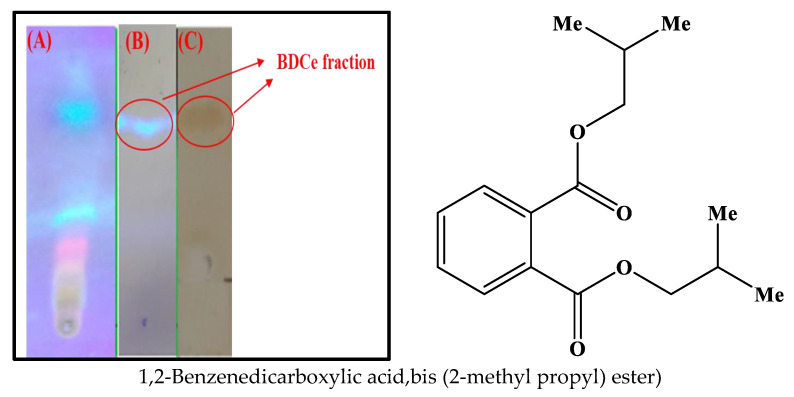
(**A**) Thin layer chromatography of *Obea* fraction of *O. bracteata*, (**B**) compound BDCe fraction after column chromatography under 365 nm light, (**C**) BDCe fraction after exposure of iodine (Solvent system = *n*-hexane: ethyl acetate (7:3)).

BDCe fraction was isolated from the *Obea* fraction of *O. bracteata* using silica gel chromatography. BDCe fraction was obtained as a colorless viscous material and gave blue fluorescence on a TLC plate when exposed to UV light (Plate I) (Figure 2). 

#### 2.2.2. Characterization and Structure Elucidation of BDCe Rraction

**Figure 3:**
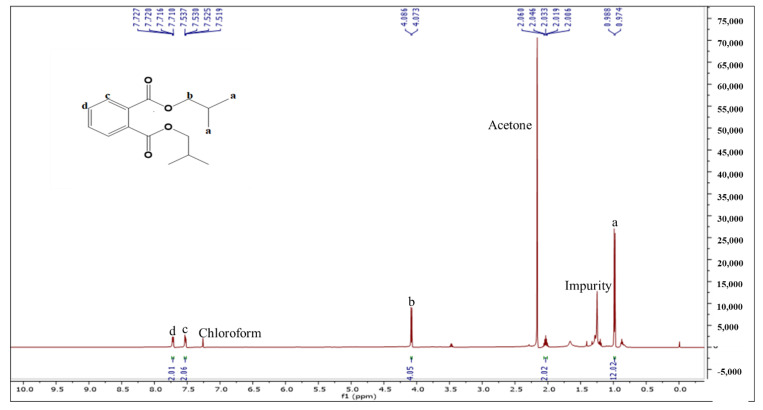
^1^H-NMR of the BDCe fraction from *Obea* fraction of *O. bracteata*.

The structure of BDCe fraction was confirmed by NMR, HR-MS, and IR analysis. ^1^H-NMR (500 MHz, CDCl_3_, ppm): two aromatic signals at δ_H_ 7.71–7.73 ppm (*m*, 2H) and δ_H_ 7.52–7.54 ppm (*m*, 2H) indicated the presence of two sets of equivalent protons. In the aliphatic region, the doublet at 0.99 (d, 12H, *J* = 6.7 Hz) showed the four equivalent methyl groups attached to (CH) methine group. Another doublet present at δ_H_ 4.08 ppm (4H, *J* = 6.7 Hz) indicated two equivalent (CH_2_) methylene groups attached with a electronegative O atom of ester. The presence of multiplet at δ_H_ 2.01–2.06 (2H) indicates the presence of two equivalent methine groups (Figure 3).

**Figure 4:**
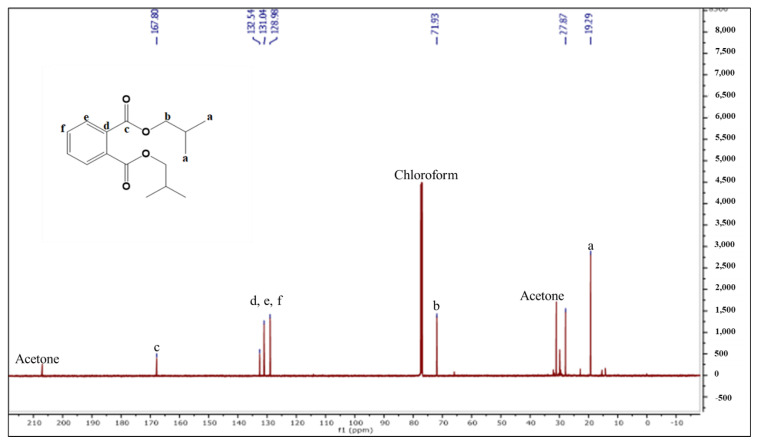
^13^C-NMR of the BDCe fraction from *Obea* fraction of *O. bracteata*.

**Figure 5:**
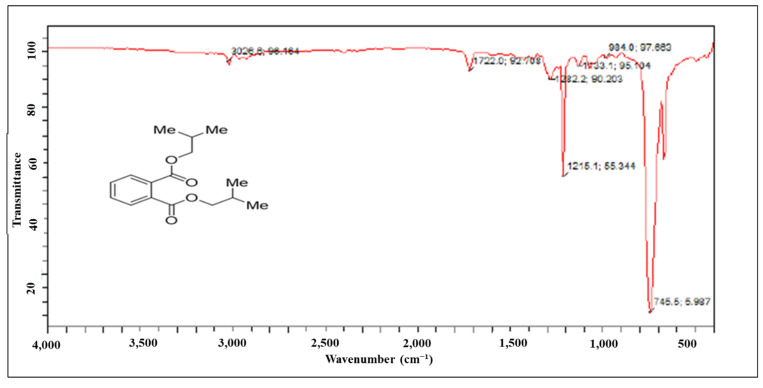
FT-IR (Fourier-transform infrared) spectrum of the BDCe fraction isolated from *Obea* fraction of *O. bracteata*.

**Figure 6:**
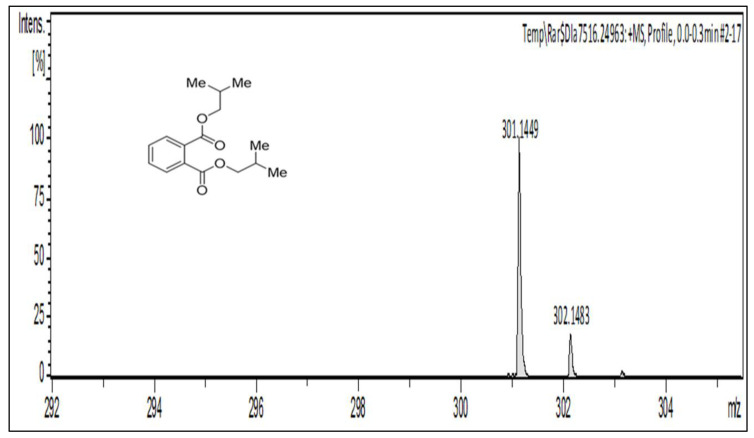
The HR-MS (high-resolution mass spectrometry) chromatogram of BDCe fraction isolated from *Obea* of *O. bracteata*.

**Figure 7:**
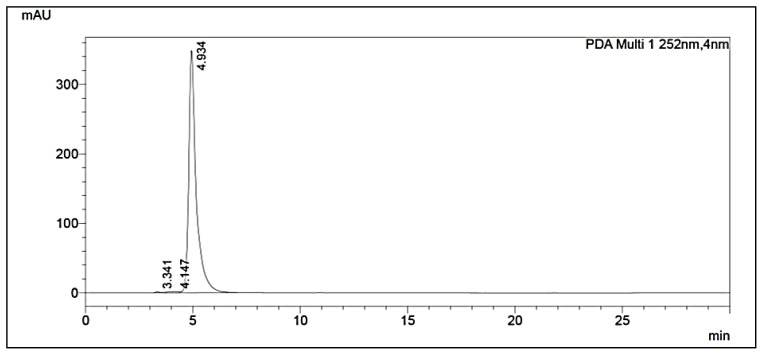
The UHPLC (Ultra High-Performance Liquid Chromatography) chromatogram of BDCe fraction isolated from *Obea* of *O. bracteata*.

^13^C-NMR (125 MHz, CDCl_3_, ppm): in the aliphatic region, the peaks at δ_C_ 19.29, 27.87, and 71.93 can be assigned to carbon atoms a, b, and c, respectively. In the aromatic region, the carbon atoms at δ_C_ 128.98 and 131.04–132.54 belong to d and e-f, respectively, and the peak at 167.8 was assigned to the ester carbon atom (Figure 4). The peak at δ_C_ 167.6 ppm in the carbon spectrum showed the presence of carbonyl ester, which was further identified by the IR spectrum (Figure 5). The HR-MS spectrum of the BDCe fraction displayed the single peak at *m*/*z* 301.1449 (M + Na^+^) (calculated 301.1410), which indicated the molecular formula to be C_16_H_22_O_4_Na^+^ at 301.1449 (Figure 6). The UHPLC analysis of the BDCe fraction showed the presence of a single peak, which was 98% pure (Figure 7).

Hence, from the above spectral data and comparison with the literature [24], the compound was identified as 1,2-benzene dicarboxylic acid, bis (2-methyl propyl) (BDCe fraction).

### 2.3. Antiproliferative Activity BDCe Fraction

**Figure 8:**
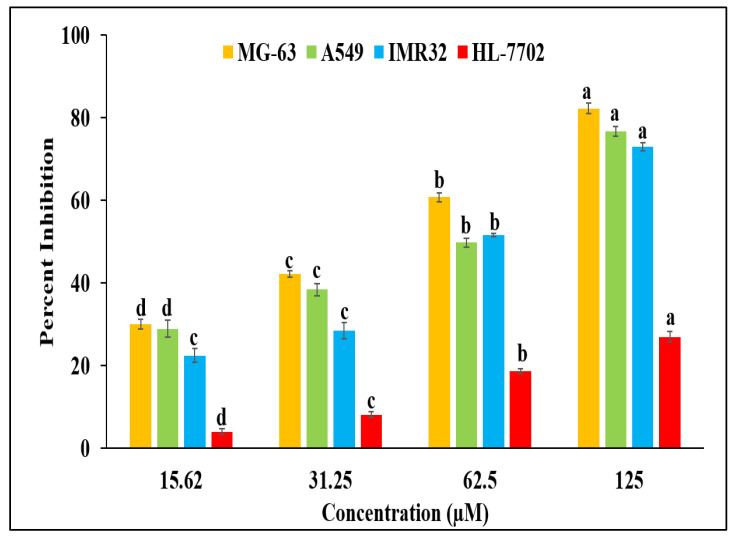
Cytotoxic potential of BDCe fraction obtained from *Obea* of *O. bracteata* on MG-63, A549, IMR32, and HL-7702 cells after 24 h of treatment. Values are expressed as Mean ± SE at a level of significance *p* ≤ 0.05. Data labels with different letters represent significant differences among them.

In the MG-63 cell line, the BDCe fraction has strong cytotoxic potential, with a GI_50_ value of 37.53 μM. The BDCe fraction also showed GI_50_ values of 56.02 μM and 47.12 μM against IMR-32 and A549, respectively (Supplementary Table S3). The standard anticancer compound (Camptothecin) showed antiproliferative potential with a GI_50_ value of 52.80 µM against MG-63 cell line. The BDCe fraction exhibited a high antiproliferative activity in MG-63 cells as compared to IMR-32 and A549 (Figure 8), which was further explored for its apoptosis mechanism of action. However, the effect of the BDCe fraction on the normal cell line (HL-7702) was minimal, which showed a GI_50_ value of 1013.35 μM (Supplementary Table S3). The HSD and F-values were found significant in all cases.

### 2.4. Morphological Change Visualized in MG-63 Cell Line

**Figure 9:**
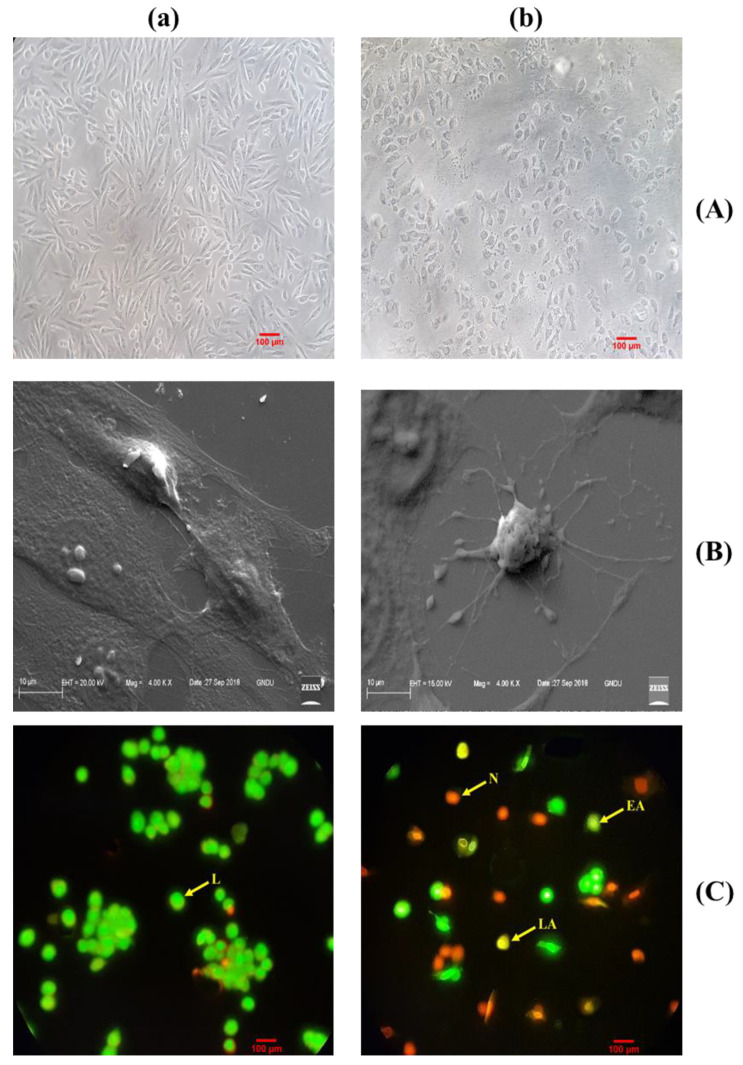

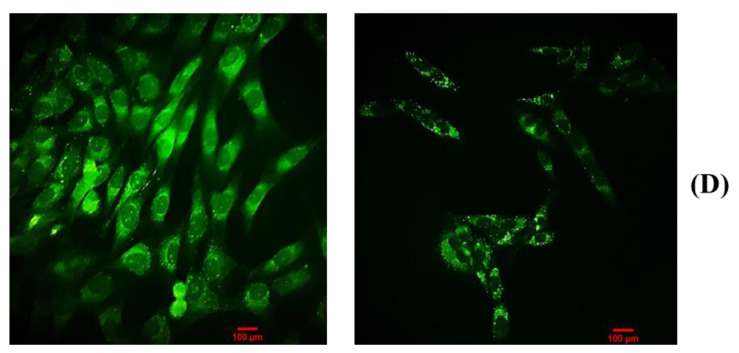
(**A**) Phase contrast images of MG-63 cells. (**B**) Scanning electron micrograph (SEM) images of MG-63 cells. (**C**) Acridine orange and ethidium bromide (AO/EtBr) staining of MG-63 cells with a fluorescence microscope. (**D**) Rhodamine-123 staining of MG-63 cells with a fluorescence microscope. (**a**) Untreated MG-63 cells, (**b**) MG-63 cells treated with BDCe fraction (37.53 μM) for 24 h. The arrow indicates live (L), early apoptosis (EA), late apoptosis (LA), and necrotic cells (N).

Morphological alterations such as the rounding-off of the cells, a gradual detachment of cells, and cell membrane blebbing were observed in MG-63 cells treated with BDCe fraction (Figure 9A).

As shown in Figure 9A(a), control untreated MG-63 cells appeared as dense multilayers. BDCe fraction-treated MG-63 cells showed alterations such as loss of contact, increased intercellular space, and rounding-off of cells.

#### 2.4.1. Scanning Electron Microscopy (SEM) Studies Confirm Apoptotic Cell Death 

SEM analysis also confirmed that the BDCe fraction induced apoptosis. SEM images revealed size reduction, membrane blebbing, and the formation of apoptotic bodies. On the other hand, untreated control cells showed intact cell morphology. This study confirms that the BDCe fraction at GI_50_ concentration induced apoptosis (Figure 9(Bb)).

#### 2.4.2. Dual AO/EtBr Staining

AO/EtBr staining showed apoptosis when MG-63 cells were treated with BDCe fraction (GI_50_) relative to the untreated control MG-63 cells. 

Untreated control cells when stained with AO/EtBr it showed green fluorescence which showed viable cells. MG-63 cells treated with GI_50_ (37.53 µM) value of BDCe fraction showed a light green nucleus, showing signs of early apoptotis of cells, cells showing an orange nucleus showed late apoptosis, and cells with a red nucleus showed signs of necrosis (Figure 9(Cb)). 

#### 2.4.3. BDCe Fraction Alter Mitochondria Membrane Potential (ΔΨm)

Loss of mitochondrial membrane integrity is one of the main events in apoptotic signaling [25]. Mitochondrial membrane depolarization was analyzed by the ability of cells to retain Rh-123 dye in untreated control and treated cells. As shown in (Figure 9(Db)), there is a decrease in the Rh-123 fluorescence intensity in MG-63 cells at 37.53 µM concentration of BDCe fraction highlighted there are losses of mitochondrial integrity.

#### 2.4.4. Hoechst Staining

**Figure 10:**
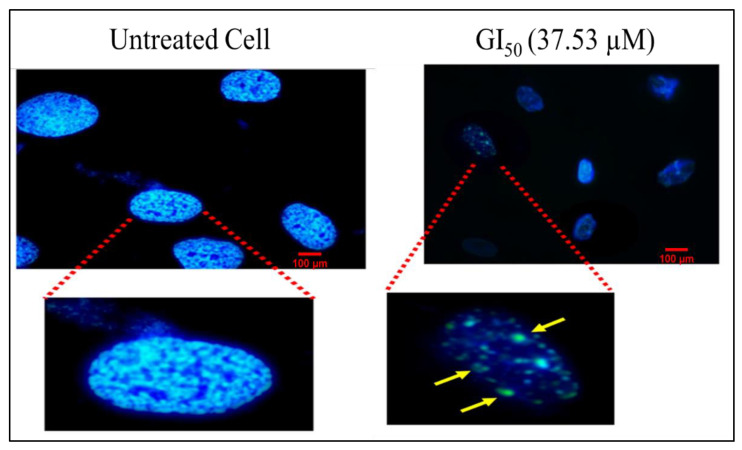
Fluorescence micrographs of Hoechst-stained MG-63 cells treated with BDCe fraction from *O. bracteata* for 24 h. Arrows show nuclear condensation/fragmentation and formation of apoptotic bodies at a magnification (100×) objective lens using oil immersion.

Hoechst 33,258 is a DNA-specific fluorescent dye that penetrates the cell and intercalates at A-T regions of DNA [26]. Typical apoptosis signs such as disintegration, shrinking, fragmented nuclei, and chromatin condensation in MG-63 cells after the treatment with BDCe fraction (37.53 µM) are seen in Figure 10. However, untreated cells showed non-apoptotic features, i.e., uniformly dispersed chromatin.

#### 2.4.5. In Vitro Cell Scratch Migration Assay

**Figure 11:**
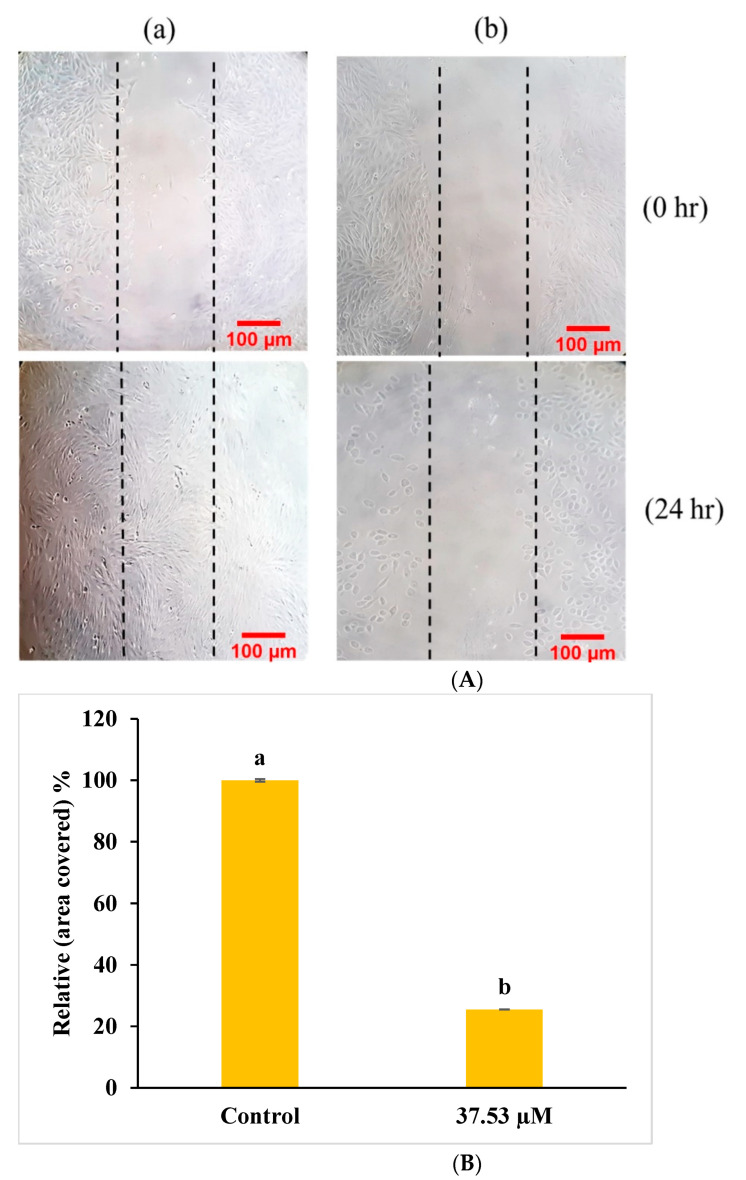
(**A**) Inhibition of the migration of MG-63 cells by BDCe fraction for 24 h.Dotted lines indicated the scratch area. (**B**) Histogram showing quantitative analysis of cell migration using alpha easeFC software. (**a**) Untreated control MG-63 cells (**b**) MG-63 cells treated with BDCe fraction (GI_50_). Values are expressed as mean ± SE at the level of significance *p* ≤ 0.05. Data labels with different letters represent significant difference among them.

The loss of migration of cells revealed the induction of apoptosis via inflammatory reactions [25]. Cell-migration assays were used to check the potential of the BDCe fraction to inhibit MG-63 cells and migrated cells into the scratch area. Phase-contrast images highlighted that the BDCe fraction has potential to inhibit the migration of MG-63 cells towards the scratch area (Figure 10). In untreated control cells, nearly 100% of MG-63 cells migrated towards the scratched area after 24 h (Figure 11A(a)).

### 2.5. Flow Cytometric Analysis

#### 2.5.1. ROS Analysis

**Figure 12:**
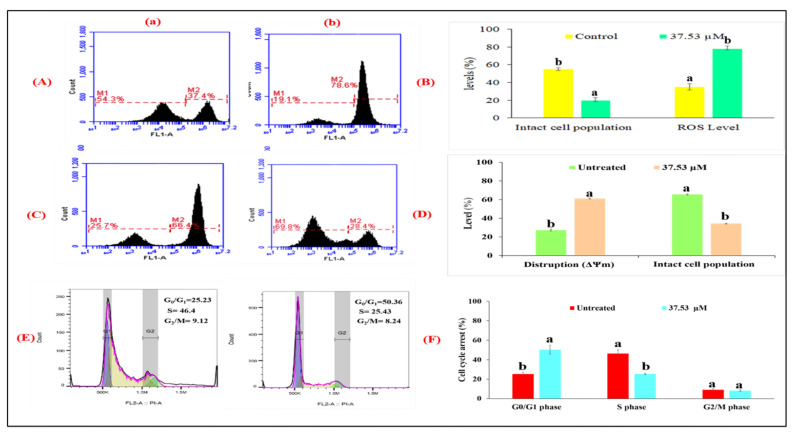
(**A**) The generation of intracellular ROS in MG-63 detected by DCFH-DA staining (M1 represents an intact cell population, and M2 represents cells with the accumulation of intracellular ROS). (**B**) Histogram showing the generation of intracellular ROS in MG-63 cells (24 h) exposed to BDCe fraction from *O. bracteata*. (**C**) The disruption of mitochondrial membrane potential (ΔΨm) in Mg-63 cells detected by staining with Rhodamine 123 (M1 represents cells with the disruption of ΔΨm and M2 represents the intact cells.). (**D**) Histogram showing disruption of mitochondrial membrane potential (ΔΨm) in MG-63 cells (24 h) exposed to BDCe fraction from *O. bracteata*. (**E**) The treatment of the BDCe fraction from *O. bracteata* (24 h) induced cell cycle arrest at the G_0_/G_1_ phase in MG-63 cells detected by a cell-cycle-analysis kit. (**F**) Histogram showing different phases of G_0_/G_1_, S, G_2_/M in MG-63 cells using a flow cytometer. (**a**) Untreated MG-63 cells, (**b**) MG-63 cells treated with BDCe fraction (37.53 µM) for 24 h. Data labels with different letters represent significant difference among them at (*p* ≤ 0.05).

ROSs play a significant role in apoptosis and in the mitochondrial-mediated pathway [27]. ROS generation was observed with a DCFH-DA probe that was deacetylated by cell esterase enzyme and oxidized by ROS into the fluorescent 2′,7′-dichlorofluorescein (DCF) [28,29]. The treatment of MG-63 cells with GI_50_ (37.53 µM) concentration of BDCe fraction resulted in an increase in the ROS generation by 78.6% as compared to untreated Mg-63 cells (37.4%) (Figure 12A). MG-63 cells with BDCe fraction displayed an increase in the DCF fluorescence, which shows the generation of ROS as well as apoptosis-inducing potential.

#### 2.5.2. Measurement of MMP (ΔΨm) Analysis

Mitochondria play a significant role in apoptosis pathways with apoptogenic factors such as the apoptosis-inducing factor, the release of cytochrome c into the cytoplasm, and the decrease in membrane potential (Ψm) [30]. An Rh-123 probe was used to detect MMP in MG-63 cells treated with BDCe fraction (37.53 µM). These results showed that MG-63 cancer cells increased the depolarization of the mitochondrial membrane by 69.8% upon exposure to BDCe fraction in comparison to untreated cells (25.7%) (Figure 12C).

#### 2.5.3. Cell Cycle Distribution

MG-63 cells treated with BDCe fraction (37.53 µM) showed significant cell–cycle delay at G_0_/G_1_ phase (50.36 ± 4.48%) as compared to the untreated cells (25.3 ± 1.84%), as shown in Figure 12E. The results showed a dose-dependent effect with a concomitant decrease in the S and G_2_/M phases.

### 2.6. Western Blotting

**Figure 13:**
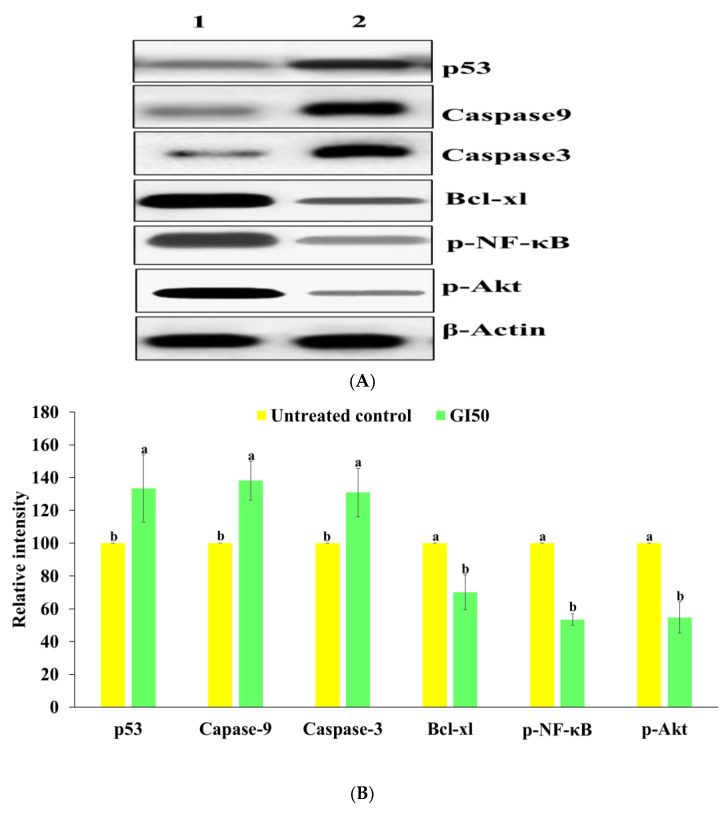
(**A**) Modulation of protein expression level of p53, Caspase-9, Caspase-3, and NF-κB, Bcl-xl, p-Akt protein induced by BDCe fraction in MG-63 cells, as detected by Western blotting. Column 1 and column 2 indicate the untreated respectively the BDCe treated MG-63 cells. (**B**) Histograms showing densitometric analysis of p53, Caspase-9, and p-NF-κB protein bands in Western blotting in BDCe fraction treated and control cells. Band density was measured and normalized to that of β-actin. Values are expressed as mean ± SE. Data labels with different letters represent significant differences among them.

Western blotting was used to identify the expression of proteins related to cell survival, proliferation, and apoptosis. There was a decrease in the expression of p-AKT, Bcl-xl, and p-NF-κB in MG-63 cells treated with BDCe fraction (37.53 µM), but the expression of Caspase 3 and Caspase 9 and p53 was upregulated in comparison to the untreated control cells (Figure 13).

### 2.7. RT-qPCR Analysis

**Figure 14:**
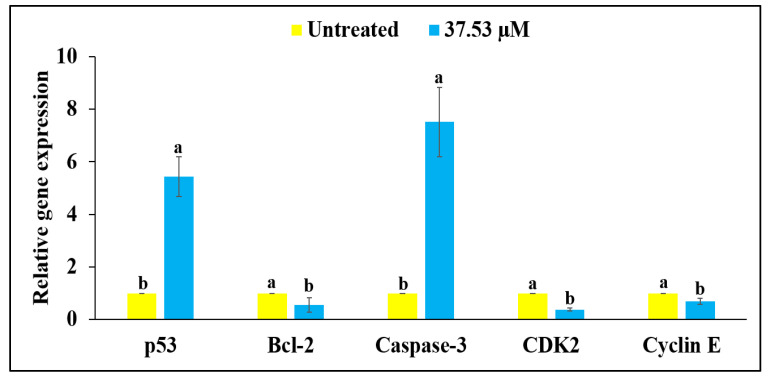
Effect of BDCe fraction from *O. bracteata* on the gene expression for p53, Bcl-2, Caspase-3, CDK2 and Cyclin E genes in MG-63 cells as detected using RT-PCR. Values are expressed as mean ± SE. Data labels with different letters represent significant differences among them at (*p* ≤ 0.05).

The RT-qPCR analysis of MG-63 cells with BDCe fraction (37.53 µM) displayed an enhancement of 5.43-fold and 7.51-fold in p53 and caspase-3 expression, respectively, but a decrease of 0.51-fold, 0.37-fold, and 0.69-fold in Bcl-2, CDK2 and Cyclin E*,* respectively, as compared to untreated MG-63 cells, as shown in Figure 14.

### 2.8. Molecular Docking

**Figure 15:**
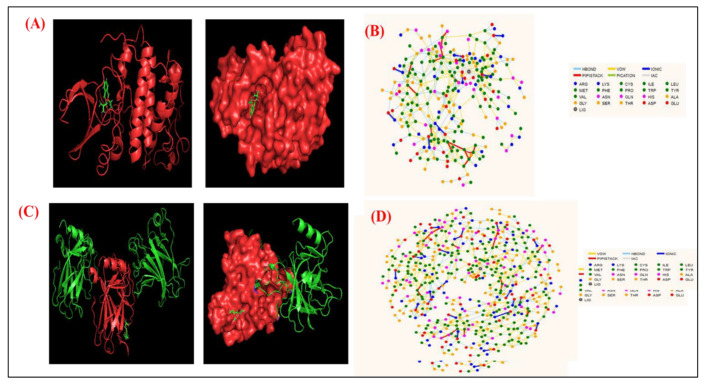
Docking conformations (PatchDock server) showing the interaction of BDCe fraction with the p53 and CDK2 binding site with minimum binding energy, (**A**) CDK-1-BDCe fraction complex with a binding energy of −133.96 kcal/mol; (**B**) CDK1-BDCe fraction RIN plot (**C**) p53- BDCe fraction with a binding energy of −151.13 kcal/mol; (**D**) p53-BDCe fraction RIN plot. RIN analysis (RING 2.0 web server) used to show interactions among proteins.

The Material PatchDock server was used for docking analysis of BDCe fraction with CDK1 and p53. We observed the docking energy for ligand-CDK-1 and ligand-p53 complex to be −133.96 kJ/mol and −151.13 kJ/mol, respectively, which indicates the stability of the docked complex (Figure 15).

To identify key residues involved in the interactions, the residue interaction network (RIN) profiles of docked complex was generated using the RING 2.0 web server. The analysis of the docked structure and the RIN plot showed that Leu 67, Phe 82, Gly 16, Ala 31, Gly 11, Phe 80, Asp 145, Ala 144, Asn 132, Gln 131, and Asp 127 amino acids were the predicted CDK1 residues that were involved in binding with the BDCe fraction in a complex structure. Similarly, we observed that Ple 232, His 233, Glu 221, Val 225, Glu 224, Gly 199, Pro 219, Thr 231, Pro 223, Asn 200, and Val 218 were the predicted p53 residues, and these showed interaction with BDCe fraction in a docked complex. (Figure 15D).

## 3. Discussion

Secondary plant metabolites play a crucial role in chemoprevention strategies. Numerous natural compounds have shown the potential of altering cellular signaling pathways due to their antioxidant, anti-metastatic, pro-apoptotic, and anti-proliferative properties [31,32]. These natural compounds specifically halt the progress of carcinogenesis by repairing DNA damage and reducing inflammation [33,34,35].

Phthalates are petrochemicals that are employed in a wide range of industrial products as plasticizers or solvents [36]. A previous report revealed that phthalate is present in various components (stems, leaves, flowers, fruits, roots, and seeds) of 60 plant species belonging to 38 families [37,38,39]. Surprisingly, several phthalates are not extensively used in industry, indicating that they may come from biosynthesis rather than contaminated soil or air [40]. Phthalates have numerous biological properties, such as anti-inflammatory, antiviral, anti-tumor, antidiabetic activity, etc., indicating their valuable potential to be explored further in capacities other than plasticizers [41]. A secondary metabolite (Di-*n*-butyl phthalate and diisobutyl phthalate) isolated from *Cladophora fracta, Croton lachynocarpus,* and *Pyrus bretschneideri* revealed that the plant has the capacity to synthesize them to some extent [42,43,44]. Kazemi [17] reported that diethyl ether extract of *Anchusa italica* (Boraginaceae) showed the presence of 34 major compounds, including diisobutyl phthalate (14.6%) and dibutyl phthalate (9.0%). The BDCe fraction effectively controls the proliferation of MG-63 cells with a concentration of 37.53 μM (GI_50_). Perveen et al. [45] revealed that the presence of Bis (2-ethylhexyl) phthalate from *Epicoccum nigrum* of *Ferula sumbul* is antiproliferative against human melanoma (SK-MEL-28 and A375P) cell lines with an IC_50_ of 12.26 µg/mL and 12.45 µg/mL, respectively. Khatiwora et al. [35] reported a bioactive secondary metabolite—dibutyl phthalate isolated from ethyl acetate extract of *Ipomoea carnea*—that showed antibacterial activity against *Klebseilla pneumonia*, *Proteus mirabilis,* and *Pseudomonas aeruginosa*. Maskovic and co-workers [46] reported that water extract of *Onosma aucheriana* has effective cytotoxic potential with GI_50_ values of 50.57, 40.34, and 25.24 µg/mL in RD (human rhabdomyosarcoma), Hep2c (human cervix carcinoma), and L2OB (murine fibroblast) cell lines, respectively. Natural compounds with antioxidant properties upsurge oxidative stress in cancer cells, disabling various pro-survival signals including ROS-scavenging mechanisms and signaling pathways suppressing cancer cell growth [47]. BDCe fraction treatment induced cell shrinkage, membrane blebbing, nuclear condensation, nuclear fragmentation, and disruption of cell membrane integrity in MG-63 cells, which is a sign of apoptosis as observed using HO 33,258, AO/Etbr, and Rh 123 dye staining. These different forms of morphological alterations in apoptotic cells are widely used for the identification and quantification of apoptosis [48,49]. Wang et al. [50] reported that human hepatocellular carcinoma (SMMC-7721) cells treated with Shikonin for 24 h significantly reduced the viability of cells and induced apoptosis features, i.e., membrane blebbing, cytoskeletal degradation, disappearance of the nucleus, vesiculation of cytoplasmic organelles, and rounding and shrinkage of cells. Furthermore, when comparing the intact untreated (control) cells to the *Obea* fraction treated cells, SEM and phase-contrast microscopy revealed apoptotic morphological characteristics. BDCe fraction exhibited a decrease in the migration of MG-63 cells as compared to untreated control cells, highlighting the anti-migratory/anti-invasive potential of the BDCe fraction. Cancer cells can easily migrate to adjacent tissue; this is a fundamental factor that plays an important role in tumor formation and metastasis [51]. Osteosarcoma cells have a high proclivity for metastasizing, which entails cell migration to distant sites [52]. In a cell migration assay, a BDCe fraction exhibited a decrease in the migration of MG-63 cells as compared to untreated control cells, highlighting the anti-migratory/anti-invasive potential of the BDCe fraction. Kundakovic et al. [53] demonstrated that *Onosma arenaria* possesses a reduced cell proliferation of human cervix adenocarcinoma cells (HeLa) and leukemia (K562) cells. Ukwubile et al. [54] reported that di–butyl phthalate isolated from ethyl acetate extract of *Melastomastrum capitatum* shows antiproliferation potential against breast cancer cell line (MCF-7) and ovarian cancer cell line (OV-7) with IC_50_ values of 22.71 µg/mL and 24.13 µg/mL, respectively. The production of reactive oxygen species (ROS) and the disruption of mitochondrial membrane potential play a key role in the induction of apoptosis via the activation of the caspase pathway [55]. The BDCe fraction from *O. bracteata* exhibited a 78.6% increase in intracellular ROS production, as evident from flow cytometer studies. Abnormal ROS generation is identified as a strong mediator of inflammation and consequential cell injury leading to apoptosis [56]. ROS generation activates apoptosis by triggering the mitochondrial-dependent apoptotic pathway and the mitogen-activated protein kinase (MAPK) pathway and induces proapoptotic signals resulting in cell death [57]. Thus, ROSs act as key signaling messengers in determining apoptosis or cell survival. Dilshara and co-authors [58] reported the subsequent inhibition of the growth of colon cancer (HCT 116) cells at the sub-G_1_ phase with the treatment of β-hydroxyisovaleryl shikonin isolated from roots of *Lithospermum erythrorhizon* (Boraginaceae) by triggering ROS production and promoting the apoptosis by activating capase8/9. The BDCe fraction successfully reduced the MMP (ΔΨm) by 69.68% at the GI_50_ concentration and delayed the growth of MG-63 cancer cells at the G_0_/G_1_ phase by 50.36%. Chan and coworkers [59] reported that triptolide (natural compound) has the potential to arrest the murine leukemia cells (WEHI-3) cells at the G_0_/G_1_ phase via the production of Ca^2+^, ROS generation, and the reduction in mitochondria membrane potential, which eventually leads to apoptosis. Kumar et al. [60] reported that ethanolic extract of *Onosma bracteata* has hepatoprotective potential against hepatic damage induced by carbon tetrachloride (CCl_4_) in male Wistar rats due to the presence of phytoconstituents in it. Kaur et al. [61] reported that Epiafzelechin isolated from ethyl acetate fraction of *Cassia fistula* showed antiproliferative activity due to increased ROS generation, decreased MMP, and G_0_/G_1_ phase arrest.

**Figure 16:**
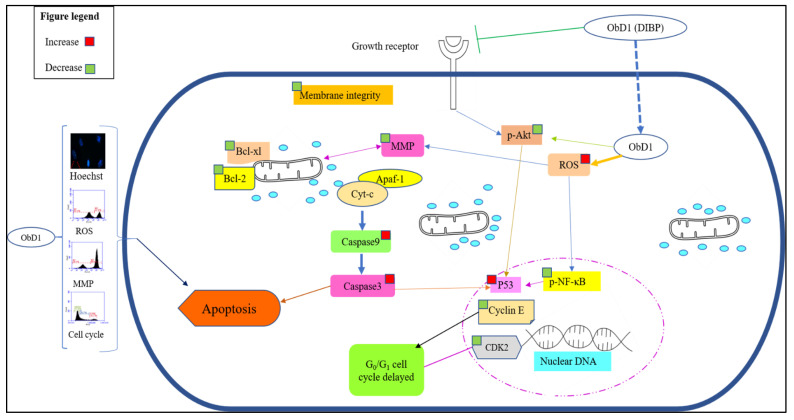
Schematic diagram showing the effect of BDCe fraction isolated from *Obea* of *Onosma bracteata*-induced apoptosis in osteosarcoma (MG-63 cells).

Dysfunction of proto-oncogenes and tumor suppressor genes are pathogenic factors for osteosarcoma (OS). Like most other malignancies, OS involves multiple oncogene activations and tumor suppressor gene mutations, including proto-oncogene c-myc, ras, fos, etc., and tumor suppressor gene p53, etc. [62]. BDCe fraction compound upregulates the activity of p53 to induce apoptosis in MG-63 cells. p53 plays an important role in inducing apoptosis; its loss results in inappropriate cell proliferation and genetic changes [63]. Modulating p53 oncogenic potential can be an effective approach to treat human cancers, as it can control many cellular functions, including cell cycle arrest, apoptosis, and senescence [64]. In the present study, BDCe fraction significantly upregulated the level of p53, caspase3, and caspase9 and downregulated the expression of p-Akt, p-NF-κB, and Bcl-xl, as indicated in Western blot studies. BDCe fraction treatment (37.53 µM) downregulated the expression of Bcl-xl showing signs of apoptosis in osteosarcoma MG-63 cells. Amalarasi and Jothi [65] reported that di-*n*-octyl-phthalate (DNOP) isolated from *Pachygone ovata* exhibited cytotoxicity against a human breast (MCF-7) cell line with a GI_50_ value of 42.47 μg/mL upregulation of via caspases 3 and 9 but downregulation of Bcl-2 gene expression. Akt supports cell proliferation by regulating the suppression of pro-apoptotic proteins and the production of cellular growth factors. Akt controls the activity of several downstream apoptosis-related proteins, such as caspase-9 and Bcl-2 family members, as well as apoptosis-interfering transcription factors, such as NF-κB [66]. Fu et al. [67] reported that Shikonin significantly inhibited inflammatory reactions via the upregulation of caspase-3 and Cox-2 and the downregulation of phosphorylated Akt and NF-κB expression in a Sprague Dawley rat model of osteoarthritis. Wang et al. [68] reported that Osthole significantly inhibited the migration and proliferation of MG-63 cells by arresting MG-63 cells at G_1_ phase via the downregulation of MMP-2, MMP-9, and p-Akt expression. BDCe fraction increased the gene expression levels of caspase3, Bcl-2, and p53 but decreased the expression of CDK2 and Cyclin E as detected in RT-qPCR studies. NF-κB is generally over-expressed in different types of cancer and is responsible for the transcription of several genes involved in tumor cell proliferation, inflammation, and metastasis [69]. Stress signals stimulate the release of cytochrome c from mitochondria, which is then associated with 47 kDa procaspase-9/Apaf-1 oligomer. This binding of procaspase-9 to apaf-1 initiates the processing and activation of procaspase-9, resulting in cleavage at Asp315 and Asp330, producing p35 and p37 subunits that amplify the apoptotic response. Cleaved caspase-9 further activates caspase-3, resulting in apoptosis [70]. Jannus et al. [71] reported that diamine-PEGylated Oleanolic Acid (OADP) showed strong anti-cancer effects in human hepatoma cells (HepG2), causing cell cycle arrest in the G_0_/G_1_ phase and the loss of the mitochondrial membrane potential (MMP). The apoptosis-induction ability of OADP was related to the upregulated expression of caspase-8, caspase-9, caspase-3, Bak, p21, and p53 and the downregulated expression of Bcl-2. Cheng et al. [72] reported that mulberry water extract had cytotoxic ability against a human liver cancer cell line (HepG2) and a human hepatocellular carcinoma cell line (Hep3B) via the activation of caspase-3, -9, and -8 and the downregulation in Bcl-2 via apoptotic-mediated pathways. Molecular docking studies also indicate that the BDCe fraction stably binds to p53 (−151.13 kJ/mol) and CDK1 (−133.96 kJ/mol). These results recommend BDCe fraction as an effective molecule with potent antiproliferative activity via apoptosis-inducing mechanisms, viz., the disruption of ΔΨm; cell cycle delayed at G_0_/G_1_ with the downregulation of Cyclin E and CDK2; the increase in the expression levels of p53, caspase-3, and caspase-9; and the decrease in the expression of Bcl-xL, p-NF-κB, Bcl-2, and p-Akt (Figure 16).

## 4. Materials and Methods

### 4.1. Chemical and Reagents

Dulbecco’s modified Eagle’s medium (DMEM), Hoechst 33,342, Fluoromount, 2′,7′-Dichlorodihydrofluorescein diacetate (DCFH-DA), and Rhodamine-123 and Fetal Bovine Serum (FBS) were purchased from Sigma (St. Louis, MO, USA). 3-(4,5-dimethylthiazol-2-yl)-2,5-diphenyl tetrazolium bromide (MTT) and trypsin were procured from Hi-media Pvt. Limited, Mumbai (India). Rabbit monoclonal Bcl-xl, p-53, p-NF-κB, Caspase3, and Caspase9 antibodies and anti-rabbit- HRP secondary antibody were obtained from Cell Signaling Technology, Danvers, MA, USA. PVDF membrane (MDI, Ambala). The RT-PCR chemical kit was purchased from Bio-Rad, CA, USA. The BD Cycletest plus DNA Kit was from BD Biosciences, San Jose, CA, USA. Chemicals and reagents of analytical (AR) grade were used to perform the experiments.

### 4.2. Plant Procurement, Identification, and Authentication of Plant Material

The plant *O. bracteata*, with accession no. GAZ-03, was purchased from the Herbal Health Research Consortium (HHRC) Pvt. Ltd. Amritsar, Punjab (India), associated with the National Medicinal Plant Board (NMPB), Ministry of AYUSH, Government of India. The plant material was deposited in the Herbarium of the Department of Botanical and Environmental Sciences, Guru Nanak Dev University, Amritsar, India (Accession no: 7576).

### 4.3. Extraction and Fractionation 

**Figure 17:**
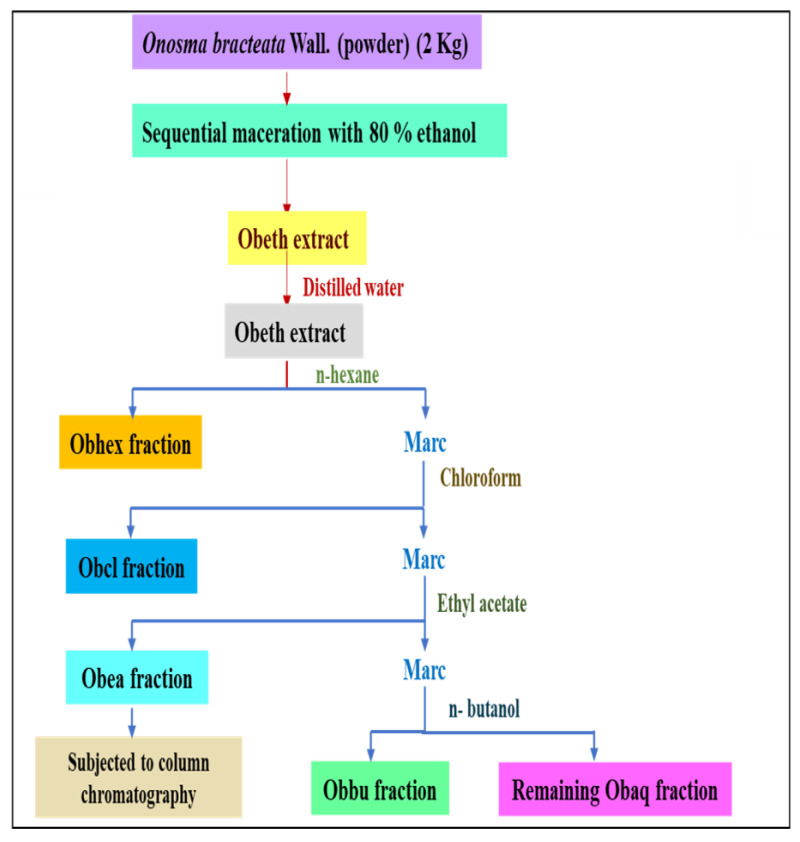
Schematic representation of extraction from *O. bracteata* using the maceration method.

The plant material was washed with distilled water and kept at 40 °C. The dried plant was coarsely ground (2 kg) and soaked in ethanol (80%) using the maceration method. The supernatant was decanted off into a flask, and ethanol was distilled using a Rotavapor (Buchi Rotavapor R-210, Switzerland) to obtain ethanolic extract (*Obeth*). Further, fractionation was performed using different organic solvents with increasing polarity, viz., *n*-hexane to yield *Obhex* fraction (6 g), chloroform to yield *Obcl* fraction (10 g), ethyl acetate to yield *Obea* (7 g), *n*-butanol to yield *Obbu* (15 g), and the remaining extract to yield *Obaq* fraction (18 g) (Figure 17).

### 4.4. Antioxidant Activity

#### 4.4.1. Superoxide Anion Radical Scavenging Assay

Superoxide radical (O_2_^•−^) is a reactive radical that functions as a precursor of reactive oxygen species (ROS) and is a mediator in oxidative chain reactions [73]. The radical scavenging potential of different fractions was investigated based on a method suggested by Nishikimi et al. [74]. Furthermore, 0.06 M nitroblue tetrazolium (NBT )and 0.156 M NADH were added to the various fraction concentrations (25–400 μg/mL), followed by 0.468 M phenazine methosulfate (PMS). After the addition of PMS, the mixture was kept for 20 min. Finally, the yellow NBT solution changed to a blue solution, and the absorbance was measured at 560 nm.

Responses in terms of percentage inhibition obtained from NBT reduction depicted by color change were represented as

$$Percent\;inhibition=\frac{OD_{C}-OD_{S}}{OD_{C}}\times 100$$

where *OD_C_* is the absorbance of control solution, *OD_S_* is the absorbance of the sample solution.

#### 4.4.2. Lipid Peroxidation Assay

The protocol proposed by Ohkawa et al. [75] was followed for the evaluation of the lipid peroxidation inhibitory potential of *O. bracteata*. For this purpose, 500 µL of lipid source (10% homogenous egg), 1000 µL of 150 mM KCL, and 1000 µL of different concentrations of fractions (25–400 µg/mL) were mixed to create the reaction mixture. To continue the lipo-oxidation, the above reaction mixture was dissolved in 100 µL of 10 mM FeCl_3_ and kept at 37 °C for 30 min. Thereafter, a 2000 µL mixture of HCl, TCA, TBA, and BHT was added, and the resultant mixture was heated at 95 °C for 1 h, followed by cooling and centrifugation. Lastly, a supernatant with a pink color was collected, and the absorbance was measured at 532 nm.

The percent inhibition (anti-lipoperoxidation activity) was calculated by the formula given below:

$$Percent\;inhibition=\frac{OD_{C}-OD_{S}}{OD_{C}}\times 100$$

where *OD_C_* is absorbance of control, *OD_S_* is absorbance of sample mixture.

### 4.5. Column Chromatography of the Obea Fraction

#### 4.5.1. Isolation of BDCe Fraction

**Figure 18:**
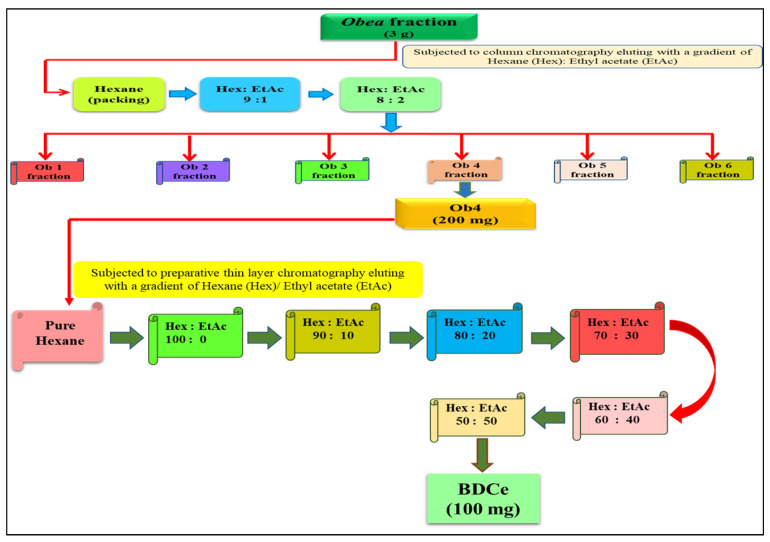
Schematic representation of BDCe fraction isolation from *O. bracteata* using column chromatography.

The slurry of *Obea* fraction (3 g) of *O. bracteata* was packed in a column with silica (mesh size 60–120) using *n*-hexane. The gradient of *n*-hexane (Hex) and ethyl acetate (EtAc) was used as the eluent. With increasing polarity, a total of 50 fractions of 50 mL each were collected and concentrated based on their thin-layer chromatography (TLC) results. Pooled fractions (Ob4) 11–15 were further subjected to preparatory TLC with a gradient of Hex: EtAc (9:1), (8:2), (7:3), (6:4), and (5:5). The blue fluorescence single spot was collected, concentrated, and lyophilized to obtain the BDCe fraction (Figure 18).

#### 4.5.2. Structure Elucidation and Characterization of the BDCe Fraction

The ^1^H and ^13^C-NMR spectra were recorded on Bruker Avance NMR 500 MHz instruments using CDCl_3_ as a solvent. The chemical shift in ppm was measured relative to TMS as the internal standard, and the coupling constant J was measured in Hz. Multiplicity is indicated as: s = singlet, d = doublet, t = triplet, m = multiplet. The HR-MS spectra were recorded on an LC-MS/MS spectrometer, HR-MS Bruker, Billerica, MA, USA. IR spectra were recorded on Agilent-FT-IR technologies, Palo Alto, CA, USA. The quantitative profiling of BDCe fractions from *O. bracteata* was carried out using a Shimadzu UHPLC Nexera system (Shimadzu, Colombia, MA, USA) equipped with a quaternary pump (LC-30AD) and a degasser. The sample was filtered using a 0.25 μm filter before analysis. The BDCe fraction was analyzed using an analytical column (C-18). For chromatographic analysis, the mobile phase was prepared by using 0.1% acetic acid and methanol solvents. Photodiode array (PDA) detector was used to detect the BDCe fraction, which was identified using UV-VIS spectrum, Waltham, MA, USA.

### 4.6. Cell Culture

IMR-32 (Human neuroblastoma), A-549 (Human alveolar basal epithelial), MG-63 (Human osteosarcoma), and HL-7702 (normal human hepatocyte) cell lines were purchased from the NCCS, Pune, India. Cells were cultured in DMEM with 10% FBS and maintained a with CO_2_ (5%) incubator at 37 °C. The cells were in the exponential phase of growth when the treatment was given.

### 4.7. MTT Assay

The cytotoxic potential of isolated compound BDCe fraction was evaluated via MTT assay based on the method prescribed by Liu et al. [76]. Suspensions of the various cell lines (8 × 10^3^ cells/0.1 mL) were seeded in 96-well microplates and incubated till confluency. Thereafter, cells were treated with a BDCe fraction using the serial dilution method. After 24 h, 20 μL of MTT was added into 96-well plates and incubated for 4 h. The supernatant SK1 µL DMSO was added to each well. Finally, the absorbance was recorded at 570 nm.

$$\%\;Growth\;inhibition=\frac{OD_{C}-OD_{S}}{OD_{C}}\times 100$$

where

*OD_C_* = untreated control;

*OD_S_* = treated sample

### 4.8. Assessment of Cells’ Microscopic Studies

For the detection of morphological changes in BDCe fraction treated MG-63 cells, were observed using phase-contrast microscopy, scanning electron microscope (SEM), and fluorescence microscopy.

#### 4.8.1. Morphological Changes of MG-63 Cells under Phase-Contrast Microscope 

MG-63 cells (2 × 10^5^/well) were cultured in 24-well plates for 24 h. After that, cells were treated with BDCe fraction for 24 h. Thereafter, cells were observed under a phase-contrast inverted microscope (Nikon Eclipse TS100, Tokyo, Japan).

#### 4.8.2. Scanning Electron Microscopy (SEM)

The SEM analysis was carried out according to the method suggested by Rello et al. [77]. MG-63 cells (4 × 10^5^ cells/well) were seeded in 6-well plates containing 12 mm circular coverslips in each well for 24 h followed by treatment of BDCe fraction for 24 h. After that, cells fixation was carried out using 4% paraformaldehyde and 2.5% glutaraldehyde. Cell dehydration was completed using chilled ethanol in a serial dilution method for 5 min each. The coverslips were mounted on the stubs with double-sided carbon tape, and silver-coating was conducted using a sputter coater (Quorum Q150R ES, Laughton, UK). Cell imaging was conducted using a scanning electron microscope (Carl Zeiss, EVO LS10, Jena, Germany).

#### 4.8.3. Fluorescence Microscopy

##### Dual Acridine Orange (AO) and Ethidium Bromide (EtBr) (AO/EtBr) Staining

AO/EtBr staining of MG-63 cells was performed as per the protocol given by Banda et al. [78]. MG-63 cells 4 × 10^5^/well were treated with BDCe fraction (GI_50_) for 24 h. Cells were collected and centrifuged at 2500 rpm for 5 min to obtain pellets. Each cell pellet was dissolved in 100 µL of 1× PBS followed by incubation for 5 min in the dark after staining with 5 µL of a mixture of AO/EtBr (60 μg/mL (acridine orange)/100 µg/mL (ethidium bromide)). The cell mixture (25 µL) was placed on a microscopic slide and covered with a coverslip. The cells were immediately imaged in a fluorescence microscope (Nikon Eclipse E200, Tokyo, Japan).

##### Determination of Mitochondrial Membrane Potential (MMP)

The mitochondrial transmembrane potential in MG-63 cells was determined after treatment with BDCe fraction using Rhodamine 123 (Rh-123) staining with fluorescence microscopy [79]. MG-63 cells (4 × 10^5^ cells/well) were cultured in 6-well plates for 24 h. After that, MG-63 cells were treated with a BDCe fraction for 24 h. Thereafter, cells were fixed with 70% ethanol followed by staining with Rh-123 solution (2 μg/mL) for 20–30 min in a CO_2_ (5%) incubator. Afterward, cells were washed thrice with 1× PBS and images were captured under a fluorescent microscope (Nikon Eclipse E200, Japan).

##### Hoechst Staining

The change in the nuclear morphology of cells was observed using Hoechst staining, as per the method suggested by Woo et al. [80]. The MG-63 cells (2 × 10^5^ cells/well) were cultured in six-well plates with 12 mm coverslips in each well. The cells were treated with a GI_50_ (37.53 µM) concentration of a BDCe fraction for 24 h. Thereafter, cells were fixed using 4% paraformaldehyde and 2.5% glutaraldehyde solution for 30 min followed by the addition of Hoechst dye (5 μg/mL) for the staining of cells. After 10 min, cells were again washed with 1× PBS. Coverslips were mounted on slides using Fluoromount. Nuclear morphological changes were observed under a Nikon eclipse Ti-2 fluorescence microscope (Nikon Corporation, Tokyo, Japan).

### 4.9. Cell Migration Assay

The migration potential of MG-63 cells treatment with BDCe fraction (GI_50_) was determined based on the method of Shah et al. [81]. MG-63 cells (5 × 10^6^ cells/well) were cultured into 6-well plates. After the confluence of the cell monolayer, a scratch was created by scraping the layer with a sterile 20 µL tip followed by washing it with 1× PBS. Images of the scrap area were taken at day zero using a Nikon Ti Eclipse in Tokyo, Japan, and plates were incubated again after being treated with BDCe fraction. After 24 h, images of the migrated cells were taken and analyzed using Image J software (version 1.52 a), National Institutes of Health, Bethesda, MD, USA.

### 4.10. Flow Cytometric Studies

#### 4.10.1. ROS Generation Analysis

The changes in ROS generation after treatment with BDCe fraction in MG-63 cells were evaluated as per the method reported by Deeb et al. [82]. The MG-63 cells (4 × 10^5^ cells/well) were cultured for 24 h in a six-well plate followed by the 37.53 µM concentration of the BDCe fraction. After 24 h, DCFH-DA (5 µM) was added to MG-63 cells and incubated for 30 min, and a pellet was obtained using centrifugation. The pellet of cells was dissolved in 1× PBS (500 μL). Finally, the level of ROS generation was measured using a flow cytometer. The obtained results were analyzed using the software provided by BD Biosciences, San Jose, CA, USA (version 1.0.264.21). 

#### 4.10.2. Mitochondria Membrane Potential (MMP) Analysis

MG-63 cells were treated with BDCe fraction (37.53 µM) in six-well plate for 24 h and further analyzed using protocol recommended by Pajaniradje et al. [83]. Rhodamine-123 (10 μg/mL) was added to the cells and kept for 30 min in the dark. Finally, the cell pellet was obtained and dissolved in 500 μL of 1× PBS. The suspension of cells was analyzed using flow cytometry for the determination of MMP.

#### 4.10.3. Cell-Cycle Phase Distribution Analysis

The BD Cycle test plus DNA Kit was used for the determination of the distribution of the cell-cycle phase in MG-63 cells. The MG-63 cells (5 × 10^5^ cells/well) were cultured and treated with BDCe fraction (37.53 µM) for 24 h. Further, cells were trypsinized and centrifugated for 5 min at 1500 rpm to obtain a pellet. Then, cells were fixed using a 70% ethanol solution followed by double-washing with 1× PBS. After fixation, the cell pellet was double-washed with 1× PBS followed by the addition of 250 μL of solution A and kept at room temperature (RT) for 10 min, and then solution B (200 μL) and solution C (200 μL) were added. The cells were analyzed by flow cytometry to determine the cell cycle phase distribution using FlowJo software (version 10.7.1).

### 4.11. Western Blotting

The expression levels of proteins involved directly in the cell signaling pathway and apoptotic proteins (p-Akt, p53, caspase3- 9, and Bcl-xl, and p-NF-κB) were analyzed by Western blotting. Firstly, MG-63 cells (5 × 10^6^) were cultured and treated with BDCe fraction (37.53 µM) for 24 hr. The cells were collected using a cell scraper, and the cell pellet was obtained by centrifugation at 1500 rpm for 5 min. For cell lysis, 150 μL of RIPA buffer was added to the cell pellet and kept on ice for 25 min and then centrifuged for 25 min. Then, the supernatant was collected and the protein concentration was quantified using the Bradford method. An equal amount of protein (40 μg) from the BDCe fraction treated and untreated MG-63 cells was resolved using SDS-PAGE and transferred to Polyvinylidene difluoride (PVDF) membrane using a wet transfer apparatus (Biorad, CA, USA). After that, the PVDF membrane was blocked using BSA (5% in TBST, 0.1% Tween-20) for 2 h at RT and incubated overnight with primary antibodies p53 (1:1000), caspase3 (1:1500), caspase9 (1:1500), p-NFκB (1:2000), and Bcl-xl (1:1000) at 4 °C. The membrane was washed thrice with TBST, and HRP-conjugated secondary antibody (1:1500) was added and the membrane was incubated for 2 h at room temperature. The blot was imaged under Image-Quant LAS 4000, GE Healthcare. Band densities were quantified with Alphaease FC Software (version 4.0). β-actin (1:1500) was used as endogenous control for stabilizing the expression of the protein of interest.

### 4.12. Quantitative Real-Time Polymerase Chain Reaction (RT-qPCR)

Total RNA was isolated with Trizol Reagent from the untreated MG-63 cells and MG-63 cells treated with BDCe fraction (37.53 µM), as per the manufacturer’s protocol. The RNA samples were dissolved with TE buffer and incubated at 60 °C for 5 min. DNA impurities were removed with DNase-I solution, and the resulting solution was incubated for 30 min at 37 °C. Finally, the quantification of RNA was performed using the Nano-Drop spectrophotometer (Thermo, Waltham, MA, USA). Further, equal concentrations of RNAs were used for cDNA preparation using the iScriptTM cDNA (Biorad) synthesis kit as per the manufacturer’s protocol. cDNA was used to perform RT-qPCR in a one-step RT-qPCR system using iQ SYBR Supermix (Biorad). The genes used as the biomarkers for RT-qPCR analysis and their primer sequences (Supplementary Table S4) were the following: p53, Bcl-2, CDK2, Cyclin E, and Caspase3. The expression of each gene was quantified by using threshold cycle method 2^−ΔΔCt^ ± SEM.

### 4.13. Molecular Docking Studies

The protein data bank (PDB) files of the solved structure of the CDK1 and p53 proteins were retrieved from the Protein Data Bank (PDB) (https://www.rcsb.org/) (accessed on 15 November 2021), a repository bank of the 3D structures of proteins and nucleic acid. The PDB file of the BDCe fraction was generated from Pymol software. The PDB file of receptors (CDK1 and p53) and a ligand (BDCe fraction) were uploaded to the PatchDock server for molecular docking analysis using cluster Root Mean Square Deviation (RMSD) at a default value of 4.0 and the protein-small ligand complex type as the analysis parameters [84]. The PatchDock server generated 10 docking complexes of p53 and CDK1 with the ligand. Finally, we selected the best docking complex based on the energy and surface area of complex molecules. To gain more insight into the docking complex, we analyzed the interacting residues of p53 and CDK1 through RIN. The BDCe fraction and CDK/p53 complex were uploaded on the RING 2.0 webserver [85]. Thereafter, we obtained the RIN profile of CDK1/p53 docking complex and analyzed the interacting key residue that directly interacted with the BDCe fraction.

### 4.14. Statistical Analysis

One-way analysis of variance (ANOVA) was used to calculate the statistical significance of all the values. The difference between the means was further compared in the high-range statistical domain (HSD) using Tukey’s test. For all experiments, the values were represented as the mean ± standard errors (SE) in triplicate values. The probability *p* ≤ 0.05 was used to demonstrate that all the values were statistically significant at a 5% level.

## 5. Conclusions

BDCe fraction exhibited strong antiproliferation potential in the MG-63 cell line (osteosarcoma) and induced apoptosis via Akt/NF-κB/p53 pathways. BDCe fraction increased ROS, decreased MMP, and delayed the cell cycle at the G_0_/G_1_ phase. This is the first report to unveil the antiproliferative potential of the BDCe fraction obtained from *O. bracteata* against osteosarcoma cell line (MG-63). This study highlights that phytoconstituents isolated from *O. bracteata,* specifically 1,2-Benzenedicarboxylic acid, bis (2-methyl propyl) ester (BDCe fraction), possess immense antiproliferative potential, which induces apoptosis in osteosarcoma cells targeting inflammatory, antiapoptotic, and proliferation markers.

## Data Availability

All datasets produced for this study are included in the article/supplementary material.

## Supplementary Materials

The following are available online at https://www.mdpi.com/article/10.3390/molecules27113478/s1, Table S1: Table S1. Antioxidant activity of extract/fractions of O. bracteata in Superoxide anion radical scavenging assay, Table S2: Antioxidant activity of extract/fractions of O. bracteata in Lipid peroxidation assay, Table S3: Cytotoxic effects of BDCe fraction on A549, IMR-32 and MG-63 cancer cell line in MTT assay, Table S4: RT-qPCR primers sequence analysis.

## Sample Availability

Samples of the compound are available from the authors.
