# Supplementary material for: Targeting Akt/NF-κB/p53 Pathway and Apoptosis Inducing Potential of 1,2-Benzenedicarboxylic Acid, Bis (2-Methyl Propyl) Ester Isolated from Onosma bracteata Wall. against Human Osteosarcoma (MG-63) Cells
Source: Molecules. 2022 May 28;27(11):3478. doi: 10.3390/molecules27113478 (PMC9182111; doi:10.3390/molecules27113478)
Supplement: Supplementary file 1 [file molecules-27-03478-s001.zip › molecules-1697166-supplementary.pdf]

**Supplementary Table S1. Antioxidant activity of extract/fractions of *O. bracteata* in Superoxide anion radical scavenging assay.**

| Conc (µg/ml) | Scavenging (%) | | | | | |
|---|---|---|---|---|---|---|
| | Rutin | Obhex | Obcl | Obea | Obbu | Obaq |
| **25** | 39.36± 2.60$^{c}$ | 16.85 ± 2.18$^{d}$ | 18.33 ± 1.21$^{d}$ | 13.77 ± 2.99$^{d}$ | 14.76 ± 3.18$^{d}$ | 15.62 ± 1.51$^{d}$ |
| **50** | 43.91± 2.93$^{c}$ | 30.87 ± 1.79$^{c}$ | 31.61 ± 2.23$^{c}$ | 30.01 ± 3.39$^{c}$ | 22.88 ± 2.10$^{d}$ | 19.43 ± 2.28$^{d}$ |
| **100** | 74.29± 1.09$^{b}$ | 50.80 ± 1.57$^{b}$ | 52.40 ± 1.82$^{b}$ | 57.81± 1.81$^{b}$ | 41.21 ± 1.25$^{c}$ | 29.64 ± 1.66$^{c}$ |
| **200** | 77.74± 1.45$^{b}$ | 59.78 ± 1.69$^{a}$ | 62.85 ± 1.98$^{a}$ | 69.61± 3.09$^{b}$ | 57.32 ± 0.68$^{b}$ | 39.11 ± 2.06$^{b}$ |
| **400** | 90.16± 1.07$^{a}$ | 66.05 ± 0.56$^{a}$ | 71.71 ± 3.25$^{a}$ | 85.36 ± 2.60$^{a}$ | 66.67 ± 1.09$^{a}$ | 59.04 ± 0.56$^{a}$ |
| **$EC_{50}$ (µg/ml)** | 46.18 | 132.23 | 114.30 | 95.12 | 160.47 | 310.91 |
| **Regression equation** | y = 19.53ln(x) - 24.88 | y = 18.37ln(x) - 39.71 | y = 19.91ln(x) - 44.31 | y = 26.37ln(x) - 70.12 | y = 19.94ln(x) - 51.28 | y = 15.36ln(x) - 38.19 |
| **r** | 0.961 | 0.980 | 0.988 | 0.991 | 0.992 | 0.967 |
| **F-ratio** | 125.18* | 155.21* | 100.34* | 106.01* | 136.74* | 102.67* |
| **HSD** | 9.27 | 7.68 | 10.26 | 13.19 | 8.78 | 8.00 |

Significance level (*$p \leq 0.05$).
Values expressed as mean ± SE.
Means with different superscripts alphabets represent significantly different values.
*Obhex* (hexane fraction); *Obcl* (chloroform fraction); *Obea* (ethyl acetate fraction); *Obbu* (butanol fraction); *Obaq* (aqueous fraction)

**Supplementary Table S2. Antioxidant activity of extract/fractions of *O. bracteata* in Lipid peroxidation assay.**

| Conc (µg/ml) | Scavenging (%) | | | | | |
|---|---|---|---|---|---|---|
| | Rutin | Obhex | Obcl | Obea | Obbu | Obaq |
| **25** | $20.06 \pm 1.36^{e}$ | $7.91 \pm 1.14^{e}$ | $11.38 \pm 1.85^{d}$ | $16.50 \pm 2.10^{e}$ | $9.79 \pm 2.18^{d}$ | $8.83 \pm 1.75^{e}$ |
| **50** | $39.12 \pm 1.76^{d}$ | $19.33 \pm 2.23^{d}$ | $19.33 \pm 2.68^{d}$ | $31.74 \pm 2.78^{d}$ | $16.28 \pm 1.06^{d}$ | $16.92 \pm 0.99^{d}$ |
| **100** | $59.28 \pm 3.48^{c}$ | $37.73 \pm 4.22^{c}$ | $39.05 \pm 3.91^{c}$ | $63.23 \pm 2.80^{c}$ | $38.74 \pm 1.69^{c}$ | $38.51 \pm 1.62^{c}$ |
| **200** | $75.09 \pm 3.73^{b}$ | $69.68 \pm 1.54^{b}$ | $68.73 \pm 1.01^{b}$ | $77.32 \pm 2.32^{b}$ | $58.55 \pm 1.51^{b}$ | $58.96 \pm 1.32^{b}$ |
| **400** | $89.88 \pm 0.83^{a}$ | $90.23 \pm 0.44^{a}$ | $90.55 \pm 2.50^{a}$ | $91.50 \pm 1.04^{a}$ | $92.30 \pm 1.19^{a}$ | $92.84 \pm 0.95^{a}$ |
| **$EC_{50}$ (µg/ml)** | 76.77 | 117.52 | 114.31 | 80.67 | 125.79 | 127.03 |
| **Regression equation** | y = 25.33ln(x) - 59.97 | y = 31.01ln(x) - 97.84 | y = 29.96ln(x) - 92.19 | y = 28.21ln(x) - 73.88 | y = 29.91ln(x) - 94.58 | y = 30.02ln(x) - 95.40 |
| **r** | 0.997 | 0.987 | 0.983 | 0.988 | 0.976 | 0.975 |
| **F-ratio** | 121.80* | 222.03* | 167.18* | 195.39* | 450.18* | 604.67* |
| **HSD** | 11.75 | 10.76 | 12.03 | 10.43 | 7.37 | 6.38 |

Significance level (*$p \leq 0.05$).
Values expressed as mean ± SE.
Means with different superscripts alphabets represent significantly different values.
*Obhex* (hexane fraction); *Obcl* (chloroform fraction); *Obea* (ethyl acetate fraction); *Obbu* (butanol fraction); *Obaq* (aqueous fraction)

**Supplementary Table S3. Cytotoxic effects of BDCe fraction on A549, IMR-32 and MG-63 cancer cell line in MTT assay.**

| **Conc. (µM)** | **Percent Inhibition (BDCe fraction)** | | | |
|---|---|---|---|---|
| | ***A549 Lung cancer cell line*** | ***IMR-32 neuroblastoma cell line*** | ***MG-63 osteosarcoma cell line*** | ***HL-7702 normal human hepatocyte cell line*** |
| **15.625** | 28.99 ± 2.09[d] | 22.52 ± 1.71[c] | 30.07 ± 1.21[d] | 4.06 ± 0.30[d] |
| **31.25** | 38.40 ± 1.45[c] | 28.50 ± 1.87[c] | 42.27 ± 0.82[c] | 8.20 ± 0.68[c] |
| **62.5** | 49.77 ± 1.11[b] | 51.58 ± 0.36[b] | 60.75 ± 1.14[b] | 18.63 ± 0.9[b] |
| **125** | 76.71 ± 1.19[a] | 72.99 ± 0.90[a] | 82.23 ± 1.24[a] | 26.97 ± 0.60[a] |
| **Regression equation** | y = 22.39ln(x) – 36.26 | 5.24ln(x) – 51.61 | y = 25.03ln(x) – 40.73 | y = 11.30ln(x) - 28.25 |
| **r** | 0.968 | 0.976 | 0.992 | 0.986 |
| **$GI_{50}$ (µM)** | 47.12 | 56.05 | 37.53 | 1013.35 |
| **Camptothecin ($GI_{50}$) (µM)** | 53.37 | 64.34 | 52.80 | 74.84 |
| **F-ratio** | 186.15* | 287.77* | 412.29* | 231.88* |
| **HSD** | 6.85 | 6.16 | 5.07 | 3.07 |

Significance level (*$p \leq 0.05$).
Values expressed as mean ± SE.
Means with different superscripts alphabets represent significantly different values.

**Supplementary Table S4. RT-qPCR primers sequence analysis.**

| S. No. | Primer Name [Acession No.] | Product Size | Oligonucleotides (5′-3′) sequence | Source |
|---|---|---|---|---|
| 1. | p53 [NM_000546.5] | 199 | Forward-TCACTGAAGACCCAGGTCCA<br>Reverse -TTGGCTGTCCCAGAATGCAA | NCBI |
| 2. | Bcl-2 [NM_000633.2] | 123 | Forward-AGTCTGGGAATCGATCTGGA<br>Reverse-GGCAACGATCCCATCAATCT | NCBI |
| 3. | Cyclin E [NM_001238.3] | 150 | Forward- GGTATCAGTGGTGCGACATAG<br>Reverse- CCAAGCTGTCTCTGTGGGTC | NCBI |
| 4. | CDK2 [NM_001798] | 180 | Forward- GGCCCTATTCCCTGGAGATTC<br>Reverse- CGTCCATCTTCATCCAGGGG | NCBI |
| 5. | β-actin [T25383] | 166 | Forward- GTCCTCTCCCAAGTCACACA<br>Reverse- GCTCATACATCTCAAGTTGGGAC | NCBI |
